# Selections

**Published:** 1817-07

**Authors:** 


### PART III.

### SELECTIONS.

—— Factorum est copia nobis.

### FOREIGN JOURNALS.

I. PHYSIOLOGY.—*Memoir on the nutritive Properties of Substances destitute of Azote.**

M. Magendie observes, in commencing this memoir, that little is known respecting the molecular action which constitutes the nutrition of animals. Its existence is incontestibly proved by the habitual excretions, the necessity of food, and by direct experiment. It is more rapid in early than advanced age, and is remarkably modified in different classes of animals; but its peculiar essence, the variations which it undergoes in each organ, and the laws by which it is regulated, are almost unknown: and the speculations of physiologists respecting it, however ingenious, exhibit only an expression of their ignorance.

Yet correct data upon the principle of nutrition are much to be desired. It is one of the most general and important phenomena in animal bodies, and many diseases seem only to be alterations of it. Hence discoveries respecting it might prove alike interesting to physiology and to medicine.

The source of the azote so abundantly contained in animal bodies forms an important and long-agitated question in the history of nutrition. By different persons, it has been regarded as the exclusive product of the aliment; of absorption from the atmosphere by the respiratory organs; or, lastly, as generated by the vital influence. But the advocates of these opinions have sought to establish them rather by reasoning than experiment; and hence the query remains undecided.

---

* *Mémoire sur les propriétés de substances qui ne contiennent pas d'Azote*, par M. F. Magendie. *Annales de Chimie et de Physique.* Septembre, 1816.

They who are hostile to the opinion that azote is derived from the aliment ingested, advance in their justification the following arguments: Herbivorous animals subsist on substances purely vegetable, and destitute of azote; and yet their organs are azoted, like those of the carnivora. The Moors, while traversing the deserts of Africa, live exclusively on gum, into the composition of which no azote enters. The Negroes and cattle of the colonies are said to feed for a long time, and even to fatten on sugar, a substance analogous in its chemical constitution to gum. Several substances, not containing azote, as the oils, fat, butter, are generally regarded as highly nutritious; and the same opinion is entertained of gum, and particularly sugar. An attempt, however, is made by M. Magendie, to controvert these positions. He asserts that the vegetables, upon which herbivorous animals feed, contain more or less of azote; that the sugar eaten by the Negroes is not perfectly pure, being commonly the sugar-cane itself, nor is it used to the exclusion of other substances; and, lastly, that the fact respecting the Moors is not clearly substantiated; and that, even if it were, the question would not be completely decided by it, since a substance, not essentially nutritive, may, for some weeks, support life. Man is known, in some regions, to subsist, during part of the year, exclusively on earth.*

In order to acquire precise notions on this subject, he determined to submit animals, for a time, to a nourishment, the chemical composition of which was rigorously determined: and the dog, as feeding equally well on animal and vegetable substances, was considered most proper for the experiment. To draw a satisfactory conclusion respecting the production of azote in the animal economy, it became obviously requisite to select for the food a substance considered nutritive, but destitute of azote. Bread was objectionable; for its gluten contains azote in abundance.

The following experiments were, therefore, instituted, and are thought, by M. Magendie, to throw light upon this important object of physiological research.

A small dog, three years old, fat and healthy, was fed exclusively, but without restriction as to quantity, on refined sugar and distilled water. For the first week, he continued well, and ate and drank with avidity. In the second, he began to waste, though eating from six to eight ounces of sugar in a day. His alvine excretions were rare and scanty, but the urinary was rather copious. In the third week, the emaciation increased; the animal became dull; his strength and appetite diminished; and there appe red successively on both eyes, in the centre of the transparent cornea, a small ulceration, which rapidly extended in depth and surface, so that the corneæ were soon perforated, and the humours esc ped from the apertures. An abundant secretion of the palpebral glands accompanied this curious phænomenon. Meanwhile, the emaciation and weakness went on; and, although the animal ate three or

* See our last *Selections*, vol. iii, page 500. Edit.

four ounces of sugar daily, he was soon incapable of deglutition or other movement. He died on the thirty-second day.—On dissection, there was a total absence of fat. The muscles were reduced more than five-sixths of their ordinary volume; and the stomach and intestines much diminished and contracted. The gall and urinary bladders were distended by their respective fluids. M. Chevreul, on examination, found in them all the characters which belong to the bile and urine of herbivorous animals; for the urine, instead of being acid like that of carnivora, was sensibly alkaline, and shewed no trace of uric acid or of the phosphates. The bile contained a considerable proportion of picromel, a peculiar character of the bile of the ox, and herbivorous animals in general. The excrements, contrary to custom, contained but little azote.

Results precisely analogous in every respect, were obtained from two similar experiments, except that, in the dog, which was the subject of the second, the corneæ did not begin to ulcerate till the twenty-fifth day, and the animal died before the humours were evacuated.

As this defect of the nutritive quality might be peculiar to sugar, it became important to ascertain whether other substances, devoid of azote, but generally considered nutritious, would produce similar effects. Two small, but young and vigorous dogs, were fed wholly on olive-oil and distilled water. They continued well about fifteen days, but afterwards suffered the same train of symptoms, as those which had lived on sugar. There was, however, no ulceration of the cornea. They both died about the thirty-sixth day. They presented, with respect to the state of the organs and the composition of the bile and urine, the same phænomena as the preceding.

Gum is another substance containing no azote, but yet accounted nutritious. With a view of obtaining direct proof that it would act like sugar and oil, several dogs were fed with it, and exhibited phænomena not sensibly differing from those just recorded.

The same experiment has been made on another dog with butter, a substance destitute of azote. The animal began to lose his flesh and strength about the fifteenth day. He died on the thirty-sixth, although, four days previously, he had been allowed meat, and eaten a quantity of it. The cornea of the right eye only was ulcerated.—Dissection here offered the same results as that of the former animals.

Although the nature of the excrements voided by these animals, announced proper digestion, M. Magendie, in order that the failure of nutrition may not be attributed to any defect in the digestive process, gave oil, gum, and sugar, separately to several dogs; and found, on opening them, that these substances were each reduced to a peculiar chyme in the stomach, and furnished an abundant chyle. That resulting from the ingestion of oil, was of a very decided milk-white appearance; while the products of the gum and sugar were transparent, opaline, and more aqueous than that of the oil.

These facts are regarded by the French physiologist as throwing doubt upon the nutritive properties attributed to oils, fat, gum, and particularly sugar. He thinks it remarkable that these substances although easy of digestion and capable of furnishing chyle, can only support life for a limited time, and yet much longer than is necessary for an animal to die of hunger. A dog, kept without food, will not live more than ten or twelve days.

In a medical point of view, it may be important to ascertain how far the influence of regimen may operate on the composition of the animal fluids, as bile and urine. Happy would it be for humanity, if, by this mean, the formation of biliary or urinary calculi could be prevented, or their increase retarded. Most urinary concretions contain a great proportion of uric acid, of the phosphates, and ammonia. Now these substances consist principally of azote. And it may reasonably be presumed, from the experiments just recorded, that by diminishing the ingestion of *azoted* substances, we might lower that proportion of principles in the urine, which give rise to the formation of calculi.

From these facts, the French physiologist considers it probable, if not positively proved, that the azote of the animal economy is principally extracted from the aliment; but, as new views are thus suggested in the treatment of one of the most terrible of human diseases, he proposes to resume the investigation on a more extended scale; and especially to ascertain whether the employment of *non-azoted* aliment in the human system would cause the uric acid and phosphates to disappear from the urine ; and whether, consequently, the formation of urinary calculi can be obviated by regimen.

Such are the experiments detailed by M. Magendie ; and such the conclusions which he draws from them : but, by the editor of one of the British Philosophical Journals,* they are represented as inconclusive, and even contradicted by notorious facts. Persons, this gentleman contends, have been found at sea, who, for several days after shipwreck, had subsisted on nothing but the sugar of the cargo ; and, again, many infants, from circumstances precluding the use of other aliment, are exclusively fed upon sugar and water.

In a note appended to his Memoir, M. Magendie answers these objections, which, he thinks, do not at all invalidate his conclusions. Man, like the dogs submitted to experiment, may subsist for some days on sugar, but cannot long survive the prohibition of other food. He then relates, on the authority of M. Moreau de Jonnès, the case of five men, who, in the year 1793, were found far out at sea, by a French man of war, clinging to the wreck of a Hamburgh packet, and who, for nine days, had subsisted wholly on sugar, and a small quantity of rum. These unfortunate persons were in such a state of exhaustion, that they with difficulty sustained removal from the wreck, and, notwithstanding every atten-

* See *Journal of Science and the Arts*, No. iii, page 133.

tion exerted to recover them, the three elder died before the vessel returned to l'Orient.

An interesting experiment on the nutritive properties of sugar is also said to have been recently made by Dr. Stark, an English physician, who lived exclusively on this substance for about a month, but was then compelled to relinquish it. He had become very weak, and swollen. His face presented red livid spots, which seemed to announce impending ulceration. He died shortly after the experiment, and is thought by persons who knew him, to have fallen a victim to it.

Some time since, a nearly similar experiment was made by an individual at Paris. Potatoes and water constituted his sole nourishment. At the end of a month his weakness was excessive, and the urinary secretion unusually abundant. On resuming *azoted* food, he recovered in a few weeks.

M. Magendie farther observes, that he has already made some trials of the treatment of urinary concretions by a diet destitute of azote. A month elapses ere the effects of this plan on the formation of gravel are perceptible. The quantity of urine is then much increased, and the body sensibly wastes. He promises to publish the results of his future observations on this subject; and he, meanwhile, entreats physicians to prosecute an inquiry, which may prove equally beneficial to physiology, animal chemistry, and medicine.—In the treatment of gravel by regimen, it is not necessary to drive the uric acid entirely from the urine, but merely to diminish its excess. Almost all, who suffer from gravel, are of a full habit, feed heartily on meat, fish and milk, bread, and other substances abounding in azote, are generally past the age of muscular activity, and take little exercise. To the abuse of this kind of food may not the excess of uric acid and the phosphates in the urine be attributed? and is it not probable, that, by a great and prolonged diminution of the proportion of these substances, and by the employment of exercise, the nature of the urine may be changed? Experience must ultimately decide; but all which M. Magendie has hitherto seen, is conformable to this doctrine.

In the ensuing number of his Journal,* the English writer returns, with redoubled vigour, to the charge. He, with some lack both of justice and courtesy, denounces the observations of the French physiologist as extremely futile, and leading to no practical or useful result. His facts are represented as scanty and insulated; his experiments ill devised; and his inferences superficial. Why, it is asked, were the experiments instituted on carnivorous rather than on herbivorous animals? What useful result can be derived from suddenly and completely changing the diet of carnivorous animals, and feeding them on butter, sugar, or gum? What conclusion can be drawn from the extinction of these "poor animals," than that they have been starved to death "novo modo?" He is recommended to prosecute his researches upon graminivorous ani-

---

* *Journal of Science and the Arts;* No. iv. page 446.

mals, and to try the effect of a gradual, instead of a sudden, change in their diet. The British Editor, lastly, contends, that the presence of azote ought not only to be sought for in the secretions and excretions of the "starved animals," but in the constitution of the muscular fibre; and he attempts to refute the assertion of M. Magendie respecting Dr. Stark's death, by an assertion equally destitute of direct evidence or support: he *knows* him to have died from no such cause.

M. Magendie, finally, in a Letter to the Editors of the *Annales de Chimie*, endeavours to parry the thrusts of the English critic, and to retort upon him: but he strikes with a feeble and indecisive hand, and ill conceals, beneath an affected smile of indifference and scorn, the pain and irritation resulting from his wound. In fact, his reply is rather fraught with empty declamation than solid and convincing argument; and the whole evinces, that his nervous system has been much shaken and deranged by the rude shock which he has sustained from the Galvanic battery of the Royal Institution.

His reason for selecting carnivorous animals in preference to herbivorous, as the subjects of experiment, was founded on the idea, that the former being more closely allied to man, the results might be susceptible of useful application to the human œconomy. He proposes, hereafter, to prosecute his researches on those of the herbivorous class.* In conclusion, he asserts, that, however man and other animals may at first suffer from an abrupt change of diet, they are never known to perish from the sole operation of this cause.

Here the matter at present rests. Between two such disputants as the author of the *Precis Elementaire de Physiologie*, and the Editor of the Journal of the Royal Institution of Great Britain, we presume not to mediate or decide: and even our opinions on this subject, if entitled to the respect which we claim not for them, are probably little calculated to serve the turn of either party. M. Magendie, although certainly prone to the error "of incorrect generalization" from imperfectly developed facts, we *know* to be an enlightened man, and a zealous and able physiologist; and, so far from considering his present researches futile and unprofitable, we exhort him to prosecute them with increased care, and vigilance, and circumspection: and, however highly we may be disposed to estimate the acquirements of the British Editor, and the value of his work, it cannot be denied that he occasionally affects a confident and dictatorial tone, but ill becoming the cautious spirit of philosophical inquiry, and which no elevation of character or talent will be found to justify. Yet it is evident, that the doctrine of the French phy-

---

* Should our pages be honoured by the perusal of M. Magendie, we would beg leave to intimate to him the propriety, not only of repeating his experiments upon *herbivorous* animals, but of trying the effect of a *sudden* and *exclusive* restriction to *such vegetable substances as avowedly contain a proportion of azote*, upon the œconomy of *carnivorous* animals. Edit.

siologist, respecting *non-azoted* alimentary substances, at present rests on very precarious ground; and that such doctrine will require for its establishment the test of more close and rigorous experiment than it has hitherto undergone. EDITORS.

---

### II. PATHOLOGY and PRACTICE.—*Memoir on a gangrenous Affection of the Mouth, peculiar to Children.**

The affection, here described by Dr. Baron, is frequent in hospitals, where many children are collected; but has hitherto been little or incorrectly observed. By different writers, it has been regarded as an effect of scurvy, or a sort of malignant pustule. The name of carbuncle, usually applied to it, is inappropriate, as it evidently constitutes a distinct and peculiar affection. Dr. Baron proposes, first, to establish the character of this fatal and interesting disease, by the recital of cases which he has observed in the *Hôpital des Enfans;* secondly, to compare it with other affections for which it may be mistaken; and, lastly, to point out the most successful mode of treatment.

1. *Character of the Disease.* Six cases are given in illustration by Dr. Baron. Of these we shall select three, the most strongly marked and interesting, as quite sufficient for our purpose, without swelling the article beyond a convenient limit.

FIRST CASE. A girl, aged six, had been, four days, suffering from small-pox, when admitted, October 17th, 1805, into the hospital. The eruption was copious, but regular in progress; yet the child continued in a state of general weakness, and had profuse diarrhœa, which survived the termination of the disease. On the 12th of November, two aphthæ appeared on the inside of the left cheek, and spread in the two following days. The cheek, on the 15th, exhibited a firm elastic swelling: the skin was pale but glistening; the pulse small and frequent; respiration somewhat tight; the discharge from the bowels abundant, mucous, and bloody. During this day, the swelling spread, and had reached, by night, the lips and left inferior eye-lid. The mouth could no longer be opened.—16th. Towards the left commissure of the lips was observed a yellow livid spot, of the size of a lentil, and not rising above the level of the skin. The general symptoms were unchanged. On the evening of that day, the spot had become an inch in diameter, and its colour black. The child was drowsy and feeble; the pulse small and rapid. Tonics, as cinchona and camphor, were internally and topically employed.—17th. The spot was converted into a gangrenous slough, two inches in diameter, and implicating the whole substance of the cheek. The adjacent parts were swollen, and the tumefaction retained its

---

* *Mémoire sur une Affection gangréneuse de la Bouche, particulière aux Enfans.* Par M. le Docteur Baron.—Bulletin de la Faculté de Médecine de Paris, 1816, No. vi.

shining appearance. The child had been delirious during the night. In the evening, the cheek was perforated by separation of part of the slough; and the patient, introducing her fingers into the orifice, tore away the remnant without evincing the slightest sensibility. The delirium continued; and it became necessary to secure her in bed. — 18th. The whole cheek was destroyed; and the interior of the mouth, which was of a black colour, exposed. The child exhaled a most offensive odour. The delirium and other symptoms continued. On the morning of the 19th she died. Her body was not examined.

Second Case. A girl, aged five, had been three months affected with curvature of the dorsal portion of the spine, attended with frequent dry cough and dyspnœa. On January 9th, 1806, she came into the hospital. General emaciation, particularly of the face, which wore an expression of suffering, dry cough, oppression, diarrhœa, and excessive weakness, were her principal symptoms. Infusion of borage with oxymel of squill, and a pectoral julep, with acetate of ammonia, were prescribed. On the 12th, aphthæ appeared in the interior of the mouth, accompanied with swelling of the right cheek: the pulse became small and frequent. The mouth was dressed with a lotion of cinchona, camphor, and muriatic acid. On the succeeding days, the swelling covered the whole right side of the face; the skin was tense, glistening, and pale, except towards the centre of the cheek, where a livid red tinge was observed. On the night of the 15th, a livid yellow spot, as large as a lentil, appeared there. On the 16th, it was become larger and black. The child was excessively feeble, and the pulse frequent and very small. On the 17th, the eschar was about two inches in diameter, and the surrounding parts much indurated and swollen. On the 18th, the interior of the mouth was seen through the slough, and the child tore away pieces of the cheek without any expression of pain: the pulse was scarcely perceptible. 19th. Oppression, stertorous respiration, loss of voice. Death at eleven o'clock, A. M.

*Dissection.*—All the soft parts, extending from the basis of the lower jaw to the zygomatic arch, and from the masseter to the right commissure of the lips, were converted into an extremely soft gangrenous mass, the centre of which was perforated by a large orifice. The gums were destroyed; and both upper and lower maxillary bones covered by a black and intimately adherent layer. The cellular membrane of the right temporal and frontal regions contained a yellow serum. A considerable quantity of serum was also effused into the sub-arachnoideal lamellar structure. The lateral ventricles (of the brain) contained about two ounces of it. Other lesions, unconnected with the disease, are not specified.

Third Case. A girl, aged four, was admitted into the hospital on the 15th of May, 1806. She was affected with measles; the eruption of which was of a somewhat livid red colour, and attended with drowsiness and smart fever. The child continued weak after her recovery; and was subsequently attacked by the itch. About the middle of July, aphthæ appeared in the interior of the mouth. *Decoction of barley with honey of roses and muriatic acid, was employed as a lotion.* On the 20th, the whole face was swollen, indurated, glistening, and presented the aspect of impending gangrene. The swelling increased the 24th, and the size of the cheeks and lips gave to the face an hideous appearance. On the 25th, a livid spot, half an inch in diameter, was observed below the inferior lip, a little to the right. The mouth exhaled a gangrenous smell, and viscid and offensive saliva oozed copiously from it. *Actual cautery was repeatedly applied to the eschar; and tonics were administered internally.* On the 26th, the slough had spread, and the swelling of the face was extreme, the lips being everted by it. *Cauterization was repeated; and the eschar covered by fomentations of cinchona;* which was also given internally in large doses. On the 27th, an eschar appeared on the left cheek, and entirely destroyed it in two days. All the gangrenous parts were converted into a black, softish, and ragged mass; and the child died on the evening of the 29th. On *dissection*, the day after, the diseased parts were seen changed into a black flabby mass, in the middle of which were yet some portions of cellular structure, not gangrenous, but containing a yellow serum. Those parts of the face, not attacked by the gangrene, contained also a similar fluid. The teeth were loose; the maxillary bones denuded, and smeared with a blackish sanies.*

The gangrenous affection of the mouth of children is never a primary disease, but attacks those who have been enfeebled by previous illness, especially the children of the poor inhabiting low and humid situations. It is frequently consequent on acute exanthemata, as measles or scarlatina, where the eruption has been irregular, or the child remains feeble after the decline of the malady. It is also seen to succeed confluent and abundantly suppurating small-pox, and mucous fevers in scrofulous subjects. Scurvy, although regarded as the disease itself, is only one of its causes. In all cases, the gangrene is invariably preceded by aphthæ on the internal surface of the cheeks and lips, or on the gums. The period of degeneration of these ulcers into gangrene varies; but, when the latter does commence, these are its symptoms and progress: The patient experiences more pain in the mouth, which exhales an offensive odour. The ulcerations put on a grey livid colour. The corresponding side of the face speedily swells; and the tumefaction gradually spreads to the lips, eye-lids, and sometimes even to the forehead and temple. The skin of the swollen parts is commonly pale, glistening, elastic, and resistent; and the peculiar aspect which they present, announces to the experienced eye the ap-

* This affection is mentioned or described by several writers, as Fabricius Hild, and Van Swieten; as also by Capdeville and Chopart in the *Mémoires de l'Academie de Chirurgie.*

proach of gangrene. Usually the point where gangrene commences, is harder than the rest, and of a livid-red colour; but this symptom does not invariably precede its invasion. After two or three days, there appears, at the point of the skin corresponding to one of the internal ulcers, a small spot, at first yellow and then black, and perfectly circular: the epidermis separates; and the whole substance of the parts covered by the spot, is gangrenous. The gangrene, extending rapidly in every direction, destroys the cheek, lips, alæ nasi, and even eye-lids; and spreading meanwhile, in the interior of the mouth, demolishes the gums. The teeth fall out; the maxillary bones perish. The structure of the soft parts, as the disease proceeds, is changed into a blackish flabby mass. The child pulls it away by pieces: hence the perforations which expose the cavity of the mouth, and disfigure the countenance. The bones are often laid completely bare, or covered only by a blackish substance; and the whole gives out an infectious smell. A viscous and fœtid saliva is constantly discharged. While these local phænomena are developing, various symptoms of general disturbance shew themselves. Among these, are smallness and frequency of pulse, some disorder of respiration, and diminution of the secreted and exhaled fluids. The digestive functions are often undisturbed, yet sometimes there is diarrhœa: those of the brain vary. There may be drowsiness, or delirium without any sleep; prostration or undue excitement of the muscular powers. The progress of the disease is rapid; its issue commonly fatal. Death usually happens from the third to the eighth day after the appearance of the livid spot. When the progress of the disease is fortunately arrested, the slough is detached in the ordinary manner; the perforations, resulting from the loss of substance, daily contract; and the dead portions of bone exfoliate.

On anatomical inspection of the victims of this disease, the gangrenous part is found completely changed into a black, soft, lacerable substance. Yet almost always in the substance of the slough are seen some portions of fatty structure, not gangrenous, but yielding a yellowish serum. A similar fluid is always effused into the cellular texture of the adjacent parts. The bones are denuded, sometimes softened: and portions of them are detached by the slightest effort. On maceration, their substance appears as though worm-eaten. An effusion of serum is commonly found in the cavity of the arachnoid membrane, or in the ventricles of the brain; but this accumulation of fluid is merely consecutive.

II. *Diagnosis of the Disease.* An affection, called *Scorbutic gangrene of the gums,* possesses considerable analogy to the disease just described. They both particularly attack children, and destroy a part of the parietes of the mouth; but the former, with a less rapid progress, invariably commences on the gums; while the latter first invades the internal surface of the cheeks and lips, and only spreads consecutively to the gums. The scorbutic gangrene, indeed, sometimes induces this disease, but then it is only the cause, and not the disease itself.

It differs also much from *carbuncle* or *malignant pustule*, which always begins externally, and successively attacks the epidermis, rete mucosum, chorion, and subjacent parts: while the gangrene, commencing on the mucous membrane, makes its way outwards to the surface. Some error or confusion, however, may arise from the disease not having been observed in its origin.

*Gangrenous aphthæ* cannot be confounded with this affection; as their progress is widely different. In the former, the gangrene is confined to, or extends little beyond, the mucous membrane.

A gangrenous disease, some years since described by the name of *Fégar* or *Fégarite*,* displays some resemblance to it, but may be distinguished, as forming in the centre of the ulcer, a hard, fungous, conical tumour, never seen in the species of gangrene here delineated.

Hence it results, that this disease is a peculiar gangrene, of invariable progress. Dr. Underwood has cursorily described it under the the tite of *Gangrenous erosion of the cheeks*.† It is probably this which the Germans distinguish by the name of *wasser-kanker*.‡ Yet some doubt arises from the assertion of these authors that it is readily cured by the application of muriatic acid; while Dr. Baron has never seen the remedy used with success. In the present state of pathological science, the name of *cancer* is utterly inapplicable; as there is no similarity in the organic characters of the two diseases.

This affection is considered by Dr. Baron to be so common to children from their lymphatic disposition; and the constant moisture which bathes the ulcerations of the mouth, as the real cause of it. At first, the disease is purely local; for the symptoms of general disturbance are only observed when the gangrene has already commenced. It is probable, that these symptoms arise from the constant deglutition of morbid saliva mingled with pus. They resemble those of putrid fever.

III. *Treatment of the Disease.* To the ulceration of the mucous membrane of the mouth, which invariably precedes the disease, our attention should be mainly directed. These should be promptly cured, especially when the child, from previous debility, seems prone to the invasion of gangrene. The mineral acids may be applied to arrest their progress; and the cavity of the mouth

---

* A barbarous term, compounded from three different languages, the Hebrew, Greek, and Arabic; and meant to designate a gangrenous affection of the mouth, common in various parts of the Continent. Edit.

† *Treatise on the Diseases of Children*, Vol. ii, page 63.

‡ See Van Swieten's Commentaries; and a Memoir of Siebert in Hufeland's Journal, December 1811, and noticed in the Bibliothèque Médicale, 1813.

be washed out with tonic acidulous lotions. On the developement of the first symptoms of gangrene, the ulcers should be touched with muriatic acid or muriate of antimony, the whole interior of the mouth be dressed with camphor and cinchona, and the same medicines be internally administered. The employment of actual cautery, in its early stage, would more certainly limit the disease; but the introduction of the hot iron into the mouth of a struggling and affrighted child is extremely difficult. When the gangrene has implicated the whole substance of the cheek and shewn itself externally, these means are rarely successful. Of thirty children observed in these circumstances by Dr. Baron, not one has escaped. Cauterization itself was useless; but he was inclined to attribute the failure to its acting only on the surface of gangrenous parts, and not on their whole substance. Hence he proposed, in the next case that should present itself, to wait till the cheek was perforated, and then apply the actual cautery upon the orifice itself; and he had soon the happiness of rescuing from death, by these means, an apparently devoted victim.

Fourth Case. A girl, aged nine, of lymphatic temperament, and feeble constitution, came into the hospital on March 3d, 1816, with pulmonary catarrh and chronic scrofulous ophthalmy. Nothing peculiar appeared in the progress of the diseases. During her recovery from the catarrh, the child was attacked by measles, which disappeared in three days after. Towards the close of April, a slight ulceration shewed itself on the inside of the left cheek. Notwithstanding the employment of an acid gargle, and the topical use of strong muriatic acid itself, the ulcer continued: and another invaded the internal surface of the lower lip. In the beginning of May, the gum was implicated in the affection, two of the inferior teeth fell out; the cheek exhibited a slight œdematous swelling; the breath became fœtid, the pulse small and frequent; and some redness and tension were observed towards the lower part of the inferior lip. Hence the approach of gangrene might be predicted. On the 13th, the ulcerations of the mouth were very extensive, and bled on the slightest touch: the breath was very offensive; the cheek swollen and glistening; the corresponding eye-lid œdematous: and there appeared on the lower lip, towards the left, a black spot, three lines in diameter, and not raised above the level of the skin. Respiration was free; the pulse small and frequent. Cinchona with acetate of ammonia, a stimulant potion, and a gargle of cinchona, camphor, honey of roses, and muriatic acid, were prescribed; and fomentations of cinchona applied to the cheek. The eschar, on the 14th, had attained the size of a crown-piece. On the 15th, it was still larger; the lower lip perforated below its middle; and the putrid slough exhaled an infectious odour. The pulse sank under pressure. 16th. The saliva flowed involuntarily from the enlarged orifice. The hot iron was now repeatedly applied to the whole surface of the perforation. The gangrene, on the 17th, had made no farther progress; and on the bottom of the ulcer, which displayed a greyish point, the muriate of antimony was applied. From the 18th to the 22d, the sloughs separated, and a portion of the lower jaw came away; but the child's strength did not fail, and she began to speak. From the 22d to the 27th, the margins of the ulcer subsided, and the sore assumed a bright scarlet colour. Soon afterwards, the extent of the ulcer sensibly diminished; and, when Dr. Baron relinquished his post at the hospital, the child's health was perfectly re-established, and the ulcer reduced to a small orifice; which, if it continue fistulous, may easily be remedied by incision and suture.

The success of cauterization, in this case, was probably owing to the complete action of the fire on the diseased parts, and its extension to those which continued sound; for the cautery, applied before perforation had taken place, did not arrest the progress of the gangrene, or prevent its fatal termination.

Might not the eschar be cut out as soon as it begins to form; and the hot iron be subsequently applied to the incised surface? Or ought we to await the natural separation of the slough? This point experience must decide. In all cases, if there be great breadth of eschar round the perforation, it should be diminished by the knife, in order to facilitate the action of the fire. The internal administration of tonics is, of course, indicated.

*Conclusions.* Dr. Baron deems himself justified in concluding, from the facts exhibited by this Memoir, that the gangrene of the mouth in children is a disease *sui generis,* and not to be confounded with others to which it may bear some resemblance;

That it is a local affection, consequent on ulceration in the interior of the mouth;

That it commences on the internal surface of the cheeks or lips, and proceeds from within outwards;

That the general symptoms are always consecutive;

And, lastly, that the most effectual mean of cure is the application of the red hot iron, when the slough is perforated.

---

### *Case of a peculiar fibrous Substance, developed on the internal Surface of the Uterus.* By Dr. Duparque.

Catharine Dupont, aged forty, married, and the mother of two children, had always menstruated regularly till 1811. In the beginning of that year, menstruation became very irregular, both in its periods, duration, and quantity. One while abundant; it was, at other times, suppressed for several months. The abdomen progressively increased in size. But this commotion, evidently the consequence of the critical period, did not sensibly affect the woman's general health. She was capable of pursuing her ordinary occupation, that of a thread-winder.

About the middle of February, a loss of blood more profuse, and longer continued than usual, induced extreme debility; and obliged the woman to apply for medical assistance. On the first of March she presented the following appearances:—Discolouration and yellowish tinge of the skin; emaciation; abdomen large as at the seventh month of pregnancy. This swelling was caused by a pyriform tumour, rising from the pelvis two inches above the umbilicus, moveable, resistent, without fluctuation, and painless on pressure. On examination, I found the cervix uteri completely obliterated; the orifice, high up, slightly dilated and apparently closed by a softish substance, which I at first took for coagulum. From passing the finger round, I judged that the uterus was distended, and that this organ formed the abdominal tumour.

The patient informed me that the abdomen was larger and more tense previously to the hæmorrhage. Since then she had felt irregular pains arising in the lumbar region, and shooting towards the pubis. They were commonly succeeded by the expulsion of coagula.

The other symptoms were, oozing of blood mixed with fœtid sanies, the smell of which precisely resembled that of the effluvia of putrid maceration water; pulse full and soft; no appetite; sleeplessness; tongue pale; urine clear.

Rest, and the employment of mucilaginous drinks produced, in a few days, cessation of the hæmorrhage; but the reddish sanious discharge, of which I have spoken, continued to flow: it was so acrid as to inflame the vulva and internal surface of the thighs.

The patient's strength gradually declined. Fever came on, with heat and dryness of the skin. Shiverings alternated with flushes of heat; and there were frequent sensations of faintness. Wine, dry frictions, and detersive injections into the vagina, were prescribed.

On the 22d of March, after pains more severe than usual, and resembling those which precede labour, there appeared at the vulva a mass of softish but tenacious flesh. On attempting to extract it, the base of the abdominal tumour sank, and violent pains were induced. Consequently these attempts were abandoned.

On the 23d, this mass projecting further between the labia, exhaled an intolerable stench. Its surface was livid, and exhibited the commencement of putrefaction. I removed with the crooked scissars, deeply introduced into the vagina, as much of the mass as I was able; and found it composed of very delicate fibrous filaments, tenacious, in general grey or whitish, by places reddish, and intimately united. It displayed a considerable degree of moisture. Here and there were found papillæ or tubercles, varying from the size of a pea to that of a large nut. These substances were of an homogenous structure, dense, close, and somewhat resembling enlarged and almost scirrhous lymphatic glands. The abdominal tumour was sensibly decreased in size.

On the 24th, a mass, equal to the preceding, descended in the same manner from the vulva; and was removed by the knife. The uterus then, inclined somewhat to the left, presented not more than half the volume which it had previously done.

The strength visibly sank: the whole right side became œdematous; the tongue dry; and the patient died at two o'clock in the morning of the 27th.

*Dissection.* The uterus was filled by a substance similar to that which I had removed: it descended through the uterine orifice, which was dilated to the size of a crown-piece, into the vagina, and was intimately adherent to the whole internal surface of the organ, except at the posterior part. This surface was covered by a greenish grey mucous layer, under which I found the mucous membrane perfectly untouched, except that it presented superiorly a superficial ulceration, apparently recent, and probably determined by the action of the acrid sanies. The posterior wall was thin, soft, and flaccid. The corresponding portion of the fibrous substance being undetached, was that which was engaged in the vagina. Wherever this substance was adherent, the uterine parietes presented a thickness of three or four lines, without any organic change in its muscular part, so that the fibrous substance appeared to be developed upon, or by a change of, the internal membrane. The hæmorrhagic blood had probably its source in the sound posterior part of the uterine parietes.

Death was probably the result of general debility brought on by the last hæmorrhage, and of the fever excited either by the putrid drain of the protruded portions of the tumour, or by the absorption of the putrid sanies which oozed from it.*

### PART IV.

* *Bulletin de l'Athenie de Medicine de Paris.* Oct. 1816.

